# ArabBert-LSTM: improving Arabic sentiment analysis based on transformer model and Long Short-Term Memory

**DOI:** 10.3389/frai.2024.1408845

**Published:** 2024-07-02

**Authors:** Wael Alosaimi, Hager Saleh, Ali A. Hamzah, Nora El-Rashidy, Abdullah Alharb, Ahmed Elaraby, Sherif Mostafa

**Affiliations:** ^1^Department of Information Technology, College of Computers and Information Technology, Taif University, Taif, Saudi Arabia; ^2^Faculty of Computers and Artificial Intelligence, South Valley University, Hurghada, Egypt; ^3^Data Science Institute, Galway University, Galway, Ireland; ^4^Atlantic Technological University, Letterkenny, Ireland; ^5^Ahram Canadian University, 6th of October City, Egypt; ^6^ML and Information Retrieval Department, Faculty of Artificial Intelligence, Kafrelsheiksh University, Kafrelsheiksh, Egypt; ^7^Department of Computer Science, Faculty of Computers and Information, South Valley University, Qena, Egypt

**Keywords:** sentiment analysis, transformer models, deep learning, machine learning, Arabic sentiment analysis, Long Short-Term Memory

## Abstract

Sentiment analysis also referred to as opinion mining, plays a significant role in automating the identification of negative, positive, or neutral sentiments expressed in textual data. The proliferation of social networks, review sites, and blogs has rendered these platforms valuable resources for mining opinions. Sentiment analysis finds applications in various domains and languages, including English and Arabic. However, Arabic presents unique challenges due to its complex morphology characterized by inflectional and derivation patterns. To effectively analyze sentiment in Arabic text, sentiment analysis techniques must account for this intricacy. This paper proposes a model designed using the transformer model and deep learning (DL) techniques. The word embedding is represented by Transformer-based Model for Arabic Language Understanding (ArabBert), and then passed to the AraBERT model. The output of AraBERT is subsequently fed into a Long Short-Term Memory (LSTM) model, followed by feedforward neural networks and an output layer. AraBERT is used to capture rich contextual information and LSTM to enhance sequence modeling and retain long-term dependencies within the text data. We compared the proposed model with machine learning (ML) algorithms and DL algorithms, as well as different vectorization techniques: term frequency-inverse document frequency (TF-IDF), ArabBert, Continuous Bag-of-Words (CBOW), and skipGrams using four Arabic benchmark datasets. Through extensive experimentation and evaluation of Arabic sentiment analysis datasets, we showcase the effectiveness of our approach. The results underscore significant improvements in sentiment analysis accuracy, highlighting the potential of leveraging transformer models for Arabic Sentiment Analysis. The outcomes of this research contribute to advancing Arabic sentiment analysis, enabling more accurate and reliable sentiment analysis in Arabic text. The findings reveal that the proposed framework exhibits exceptional performance in sentiment classification, achieving an impressive accuracy rate of over 97%.

## 1 Introduction

Sentiment analysis, often known as opinion mining, is a branch of natural language processing (NLP) focused on extracting subjective information from textual data (Solangi et al., 2018). It involves the analysis and understanding of attitudes, sentiments, and feelings conveyed through spoken or written language (Liu, 2010). With the exponential growth of social media platforms, online review sites, and blogs, there is an enormous volume of user-generated content that contains valuable insights into people's opinions and emotions. Sentiment analysis approaches aim to extract and classify sentiments as positive or negative, providing valuable insights for various applications such as market research, brand monitoring, and customer feedback analysis (Gandhi et al., 2023).

The primary goal of sentiment analysis is to computationally understand and interpret the subjective information conveyed in a text, allowing for quantitative analysis of sentiments on a large scale (Argamon et al., 2009; Li and Hovy, 2017). By analyzing sentiment, organizations can gain a deeper understanding of public perception, track brand reputation, identify emerging trends, and make data-driven decisions. Sentiment analysis encompasses a wide range of approaches and methodologies, including ML and DL algorithms that extract sentiment-related features from text (Yue et al., 2019).

Due to its significant influence on international politics and the global economy, the Arab world has recently garnered attention worldwide. Opinion mining on various topics, including politics, market fluctuations, and oil and gas prices, has become a focal point. This trend aligns with the proliferation of social media platforms, leading to a notable increase in the number of Arabic writings available online (Zaidan and Callison-Burch, 2014). This presents a unique challenge for the Arabic language. Our objective is to identify transformation-based methods that yield better and more profound results than current approaches for analyzing and understanding sentiments in Arabic (Abdul-Mageed et al., 2023).

Despite the importance of using ML in NLP, it faces several challenges, including contextual understanding, handling multilingual sentiment, and a lack of domain adaptation. To tackle these obstacles, DL plays a crucial role in obtaining data representations and extracting significant insights during the learning journey. DL, an advanced form of ML, surpasses conventional ML models in performance. Approaches rooted in DL have proven to be highly effective in sentiment analysis thanks to their multi-layered architecture, which enables the extraction of profound features and patterns from extensive textual data (Wadawadagi and Pagi, 2020; Yadav and Vishwakarma, 2020; Ahmed M. et al., 2021).

Lately, transformer models have become increasingly popular due to their exceptional results, primarily attributed to their attention mechanisms and contextual understanding (Mishev et al., 2020). The decision between these approaches is determined by several criteria, including computer resources, training datasets, and SA task (Naseem et al., 2020) requirements. Advances in NLP have had a transformational impact on pre-trained models. These models possess a remarkable advantage in providing hundreds of millions of parameters, thereby enhancing the learning process. Among these models, BERT stands out as one of the most advanced, demonstrating noteworthy advancements in performance. It can capture contextual relationships between words in a sentence and relies on self-attention mechanisms. They efficiently capture long-range dependencies and enable parallel processing by simultaneously analyzing the entire sequence (Habimana et al., 2020).

The attention mechanism in the transformer permits dealing with each word in the sequences. This mechanism allows for the assignment of different weights to each part of the input during the learning process (Vaswani et al., 2017). It calculates weights based on the similarity between the current word or token and the other words or tokens in the sequence. The inclusion of attention mechanisms in the transformer model has revolutionized the treatment of long sequential data (Liu et al., 2022). This advancement has not only led to the widespread utilization of the transformer model across different tasks but has also had a significant impact on model performance. By efficiently capturing comprehensive context and global dependencies, the transformer model has achieved substantial enhancements in accuracy and result quality (Santana and Colombini, 2021).

The main objective of this research is to propose AraBERT-LSTM that integrates the advantages of the transformer model and LSTM model for Arabic sentiment analysis that encompasses four benchmark datasets, producing significant results as its primary outcome. AraBERT is used to capture rich contextual information and LSTM to enhance sequence modeling and retain long-term dependencies within the text data.

The contribution of this research work can be summarized as listed below:

- Proposing model based on a transformer model and LSTM, named AraBERT-LSTM. The model includes the AraBERT model, an LSTM layer, feedforward neural networks, and an output layer.
- Exploring the benefits of utilizing preprocessing techniques specifically tailored for Arabic text, which include tokenization, punctuation and stop words removal, elimination of special characters and digits, as well as normalization and stemming, to enhance the quality of the data.
- To extract relevant insights from the data, three different word embedding approaches are employed: CBOW, SkipGram, and AraBERT. These strategies enable us to effectively capture the underlying semantic information in the text.
- The proposed model is compared against multiple ML models (DT, RF, LR, KNN, NB), as well as DL models (GRU, LSTM). Results consistently demonstrate its superior performance, achieving higher accuracy rates across benchmark datasets. This evaluation is conducted using SS2030, ASTC, and Main-AHS datasets, establishing its superiority over existing models.

This paper is organized as follows: In Section 2, we provide an overview of existing research in sentiment analysis. Section 3 introduces our proposed framework, presenting its design and methodology. The experimental results are shown in Section 4, which also assesses how well our suggested model performs in comparison to baseline techniques. In Section 5, we bring the work to a close by providing a summary of our main conclusions.

## 2 Related work

Sentiment analysis is an important field that helps companies and organizations analyze customers' opinions from different sources of data on the internet, such as social media and blogs. Different techniques have been applied to extract and analyze opinions: ML models and DL models.

### 2.1 Arabic sentiment analysis using the ML models

This subsection presents related work that have been applied ML models for Arabic sentence analysis. In Musleh et al. (2022), the authors applied ML models: SVM, AdaBoost, RF, LR, and KNN to an Arabic dataset. In terms of accuracy, the RF algorithm outperformed the others. The author of Alharbi and Qamar (2021) used KNN, RF, SVM, LR, and NB on reviews written in Arabic about cafes and restaurants in the Saudi Arabian province of Qassim. Results show that the SVM algorithm provides the best accuracy. A discriminative multinomial Naïve Bayes (DMNB) model was developed by the author in AlSalman (2020) to categorize Arabic tweets into positive and negative polarities. DMNB and alternative ML models were contrasted. The DMNB model has the maximum accuracy, according to the findings. In Alyami and Olatunji (2020), the SVM model was applied to multiple social topics in Saudi Arabia using Twitter data collected by the author. Based on the results, the SVM algorithm achieved the highest accuracy. In Almouzini et al. (2019), the dataset was subjected to classification methods utilizing RF, AdaBoost, Liblinear, and NB algorithms. Both the CES-D and the Patient Health Questionnaire (PHQ-9) were employed to diagnose depressive symptoms among Arabic tweeters. The linear algorithm offers the highest accuracy when compared to other algorithms.

The authors of Elshakankery and Ahmed (2019) propose hybrid models to combine lexicon-based SVM, RNN, and LR models to enhance Arabic sentiment analysis performance. The model was tested on five datasets, and LR produced the best results in terms of accuracy. The author used KNN, SVM, NB, DT, and Bayes networks to analyze the ABSA of hotel reviews written in Arabic in Al-Smadi et al. (2019). SVM proved its accuracy when compared to other algorithms. Four Arabic SA datasets were subjected to LR, KNN, and DT by the authors in Bolbol and Maghari (2020). When compared to the other classifiers, the LR produced datasets with a higher accuracy rate. After creating the feature vector in Ahmed D. et al. (2021), using TF-IDF, the authors applied LR, SVM, RF, NB, and K-NN. The best accuracy performance was presented by SVM. In El-Masri et al. (2017), the author presented tools that analyze sentiment in Arabic text. They applied many steps: pre-processing text, feature extraction based on n-gram and lexicon-based methods, and finally, ML models. According to the findings, NB achieved the best performance.

### 2.2 Arabic sentiment analysis using DL models

This subsection presents related work that has applied DL models for Arabic sentence analysis. The authors of Elhassan et al. (2023), used LSTM, a hybrid CNN-LSTM, and convolutional neural networks (CNNs) to predict Arabic sentiment analysis. Word2Vec and fastText were employed as word embeddings. Large-Scale Arabic Book Reviews (LARB) is a database of book reviews, whereas Hotel Arabic Reviews Dataset (HARD) is a database of hotel reviews. One CNN layer and two LSTM layers make up the hybrid DL model that the authors of Ombabi et al. (2020) proposed. After learning the features using CNN and LSTM, the final prediction was made using SVM. The word embedding model FastText was applied. The Arabic sentiment analysis tweets dataset was constructed by the authors in Alyami and Olatunji (2020) in order to categorize texts' sentiments and opinions. They applied SVM using N-grams feature extraction. Accuracy achieved the highest performance using the SVM model. The authors of Oussous et al. (2020) used CNN, LSTM, and DL models to enhance the accuracy of ASA prediction. They employed multiple pre-processing techniques, such as stop words, tokenization, normalization, and stemming. The outcomes of the experiment verified that DL outperformed NB, SVM, and maximum entropy in terms of results. In Alayba et al. (2018), the authors studied the effect of applying Word2Vec on Arabic text. They applied different ML and CNN models to Arabic tweets about health services (Main-AHS). The findings show that CNN models enhanced sentiment classification accuracy. The authors in Al Omari et al. (2019), proposed and analyzed a hybrid CNN-LSTM model with ML models. They employed the word2vec word embedding to convey the features. CNN-LSTM performed the best on Main-AHS data. In Dahou et al. (2016), the authors employed CNN models for several types of Arabic data in diverse fields. Word2vec was utilized to create the word embedding. CNN-LSTM delivered the best results. In Alwehaibi and Roy (2018), the authors propose a hybrid LSTM-RNN model that combines LSTM with RNN for analyzing Arabic sentiment. Their research examined the effects of different pre-trained word embeddings on DL models. In Al-Twairesh et al. (2017), the authors presented a dataset of health services from Twitter in Arabic. The tweets were annotated into good and negative tweets. A health dataset was analyzed using NB, SVM, LR, and CNN. The authors of Omara et al. (2018), proposed contrasting deep CNN models with several ML techniques, including LR, SVM, and NB. A SA dataset is combined to create a large-scale dataset for training neural networks. This dataset collects thoughts in several Arabic formats (Modern Standard, Dialectal) from various domains. In Almouzini et al. (2019), An LSTM bidirectional network (BiLSTM) is investigated for Arabic sentiment analysis. DL and ML models were trained and evaluated using six Arabic datasets. In comparison with DL and ML models, their model performed best. The authors of Saleh et al. (2022), presented stacking DL models that merge the pre-trained models RNN, LSTM, and GRU with metal-learner to improve SA performance. Using three benchmarks from the Arabic SA dataset, they compared their proposed model against DL and ML models.

## 3 Methodology

**Figure 1:**
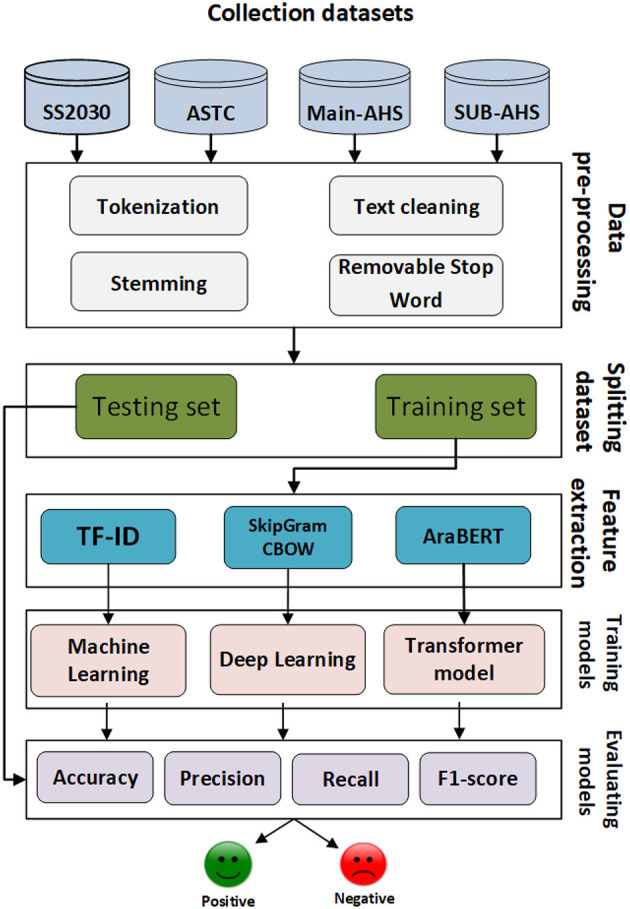
The main steps of the Arabic sentiment analysis framework.

The proposed framework consists of three main approaches: a ML approach, DL approach, the proposed model based on AraBERT and LSTM for predicting Arabic sentiment analysis using four datasets. Firstly, the Arabic sentences were pre-processed to eliminate unnecessary tokens and symbols. Then, each dataset was split into training and testing sets. TF-IDF, AraBERT, CBOW, and SkipGram were employed to convert texts into vectors. Afterward, models were trained using the training vectors and evaluated using the testing vectors. The main steps of the Arabic sentiment analysis framework as shown in Figure 1.

### 3.1 Collection datasets

This section describes the datasets used to train and evaluate models.

- Arabic Sentiment Analysis Dataset (SS2030):
  To conduct Arabic SA, SS2030 was constructed using Saudi tweets discussing various topics and events (Alyami and Olatunji, 2020). Four thousand and nine tweets total from the created dataset have been manually classified as positive or negative. Based on specific keywords linked to social concerns that were hotly disputed in Saudi society, tweets were retrieved. This dataset aims to ascertain how political reforms and social developments have affected Arabic societies.
- Arabic Sentiment Twitter Corpus (ASTC):
  The purpose of the ASTC dataset was to gather Arabic sentiment corpus so that researchers could look into DL techniques for Arabic sentiment analysis. this dataset was gathered in April 2019. It has 56,793 Arabic tweets with labels—both positive and negative— annotated.
- Arabic Health Services Dataset (Main-AHS and Sub-AHS):
  The Twitter dataset (Alayba et al., 2017) was gathered in 2016 by utilizing the Twitter API and keyword-based health-related searches. There are a total of 2,026 tweets in Main-AHS and 1,733 tweets in Sub-AHS. Positive and negative sentiments can be identified in these tweets.

### 3.2 Data pre-processing

Different data pre-processing steps are applied to text data to enhance quality of data and improve results.

- Tokenization in text preprocessing involves dividing a text into meaningful parts. Tokens are meaningful pieces of language, such as words, phrases, or other significant units. As a result, tokenization is a type of text segmentation. Sets of words are the outcome of this procedure. Among the tokenization options provided in this study is the Natural Language Toolkit (NLTK) library tokenization (Hardeniya et al., 2016).
- Data Cleaning includes: This process involves removing unnecessary details like:
  - URLs, hashtags, and user mentions are eliminated. This phase includes removing URL links (such as http://twitter.com), special characters (like RT, which stands for “retweet”), hashtags (for example, “#Climate Summit2020”) and user mention (like @Allawihamzah).
  - Deleting all punctuation
  - Deleting commercial and non-Arabic tweets such as: English letter
- Stemming: The stemming process involves creating base or root word variations. Simply put, it reduces a base word to its stem term to make the look-up process shorter and more understandable (Oussous et al., 2019). Arabic words are created from sets of roots that highlight the basic meaning of the word, along with additional suffixes that change the sound of the word. In this study, we use one of root-extraction methods which is Information Sciences Research Institute (ISRI) Arabic stemmer (Taghva et al., 2005). ISRI (Root Extraction Tool), which is a mechanism to extract roots without a root dictionary compared to Khoja stemmer. The basic objective of ISRI is to extract the minimum representation of a given word. The summary of the use ISRI algorithm in this study is as follows:
  - Eliminate the vowel-representing diacritical marks.
  - Normalize the hamza, which can be found with different letters in multiple different forms, to ensure these words have the same root, such as يأكل (he is eating) and أوكل (it is eaten) in a single form. In addition to normalizing special letters إ أ آ to ا
  - Eliminating words that have less than three letters to avoid ambiguous stems.
  - Applying the rest details of the algorithm steps to get the Arabic stemmed text without suffixes and prefixes (Taghva et al., 2005).
- Stop word removal is the most often utilized pre-processing technique in NLP applications. The primary goal of stop word removal is to eliminate frequently used terms in all of the corpus's documents. In other hand, Stop words are any words that appear repeatedly but have no impact on the content or meaning e.g., conjunctions, pronouns, prepositions such as مثل - ذلك - حيث - لدى - الا عن - ب- إلى - لنا - فقط - الذي Alrefaie (2019).

### 3.3 Splitting datasets

Every dataset is divided into 80% training and 20 %testing sets. To train ML, DL, and transformer models, utilize the training set. Models are assessed on a testing set.

### 3.4 Feature extraction methods

- For ML models, Term Frequency–Inverse Document Frequency (TF-IDF) with N-gram is used to build the feature matrix. TF-IDF feature extraction is a widely used statistical method in NLP and information retrieval. A corpus is a collection of documents that measure the importance of a given term within that collection (Rahman et al., 2020; Bountakas et al., 2021).
- For DL models, Word embedding is a technique that involves mapping each word to a low-dimensional vector within a d-dimensional space, as discussed in Mojumder's study (Mojumder et al., 2020). One prominent example of word embedding is Word2Vec, a neural network-based linguistic model that employs two training architectures: Continuous Bag-of-Words (CBOW) and Skip-Gram. Mikolov et al. (2013) introduced these approaches, where word co-occurrence statistics are utilized to construct word embeddings. CBOW operates similarly to a Feedforward Neural Network (FNN), predicting the target word based on words within a given context window. In contrast, Skip-Gram predicts context words based on a target word. Both methods optimize word embedding vectors by making predictions on a set of samples. This embedding process is crucial for enhancing NLP tasks through the efficient representation of words in a continuous low-dimensional space (Jiao and Zhang, 2021).
- For transformer model, Transformer-based Model for Arabic Language Understanding (ArabBert) is used to present a contextualized word embedding model specifically designed for the Arabic language. AraBERT is made up of an encoder with several layers of self-attention mechanisms that are specially made for processing Arabic language data (Antoun et al., 2020; Alammary, 2022). It developed by A group of researchers from the University of Maryland in the United States and King Abdulaziz University in Saudi Arabia. It is a transformer-based model that captures the contextual links between words in a sentence using a self-attention mechanism (Rahali and Akhloufi, 2023). It is pre-trained on a massive amount of unlabeled text data, facilitating its learning of the language's language features and statistical trends (Alshaikh et al., 2023). AraBERT employed BaseBERT configuration with the advantage of word segmentation into stems, prefxes, and sufxes to overcome the lexical sparsity of Arabic words. After that, the segmented pre-training dataset is used to train a sentence piece in unigram mode (Total vocabulary size: ~60 K tokens). This version of AraBERT is called (AraBERTv0.2) while (AraBERTv0.1) is created by using a non-segmented text to train sentence pieces, and it contains 64 k tokens in its vocabulary. We, therefore, employed (AraBERTv0.2). It is based on Bidirectional Encoder Representations from Transformers (BERT) that was introduced by Google in 2018 pre-trained on a large corpus of unlabeled text data. Vaswani et al. (2017) and Gao et al. (2019) high-level overview of BERT process.
- BERT is an attention mechanism that reads text input using an encoder and produces a task prediction using a decoder. It can generate a language representation model by using only the encoder portion. A single sentence or two sentences can be represented as a series of tokens by the task-specific BERT design. The input representation of a token is created by adding the token, segment, and position embedding that corresponds to it.
- Additional tokens are appended to the tokenized sentence at its start ([CLS]) and end ([SEP]). By adding one or more layers on top of BERT and training all of the levels simultaneously, BERT can be adjusted to a downstream NLP goal (Vaswani et al., 2017; Gao et al., 2019).
- A fully connected layer is then connected at the [CLS] position of the final encoder layer, and the classification of sentences or sentence pairs is finished by a softmax layer.

### 3.5 Machine learning approach

This section presents define about LR, RF, DT, SVM and KNN:

- Logistic Regression (LR) describes the relation between a binary or dichotomous outcome (response variable) and a set of independent variables (Das, 2021).
- Decision Tree (DT) is a flowchart-like structure where internal nodes are represented by rectangles and leaf nodes by ovals (Priyam et al., 2013).
- Support Vector Machine (SVM) is developed based on the structural risk minimization theory. The best-separating hyperplane is found through mapping input vectors to a high-dimensional feature space (Vapnik, 1999; Peng et al., 2013).
- K-nearest Neighbor (KNN) classifies objects by using the most recent training samples in the feature space (Peng et al., 2013).
- Random Forest (RF) is a supervised ML technique that can be used to solve classification and regression problems. Through mixing N decision trees and building each tree with an out-of-bag sample for classification, this approach can tackle the missing value problem (Roy et al., 2020).
- Naïve Bayes (NB) is a well-known data mining classification technique that assumes that all attributes are independent of one another and calculates the likelihood that a fresh example belongs to a given class. This assumption is motivated by the use of training data to estimate multivariate probabilities (Chen et al., 2020).

### 3.6 Deep learning models approach

Long Short-Term Memory (LSTM), and Gated Recurrent Unit (GRU) are employed with two embedding words: Skip-Gram and CBOW.

- LSTM is an extension of the Recurrent Neural Network (RNN) commonly employed in deep learning. It excels over RNN in capturing long-term dependencies, making it ideal for tasks involving sequence prediction (Vennerød et al., 2021). With memory cells retaining information over time, LSTM utilizes feedback connections based on them to interpret entire data sequences rather than individual data points (Lindemann et al., 2021). The gating mechanisms of LSTM regulate information flow, allowing the network to selectively read, write, and erase data from memory. The input gate determines how much new input can be stored in the cell state, while the forget gate decides which unimportant data should be removed from the cell state. The hidden state, the output of LSTM at a specific time step, incorporates data from previous time steps and is used to compute the output of the current time step (Lindemann et al., 2021).
- GRU is a type of DL architecture commonly employed for modeling sequential data. The GRU architecture comprises several essential components. The update gate, taking into account the current input and the preceding hidden state, controls the amount of information from the previous hidden state to be transferred to the current time step (Chung et al., 2015). Determining the extent to which the previous hidden state should be forgotten, thus ceasing to influence the current time step, is the role of the reset gate, which considers both the previous hidden state and the current input. The calculation of the candidate activation involves the reset gate, the previous hidden state, and the current input. Subsequently, the hidden state constitutes the output of the GRU at a given time step (Dey and Salem, 2017).

### 3.7 The proposed model

**Figure 2:**
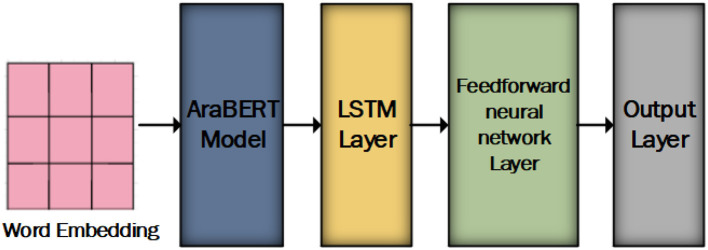
The proposed model for the Arabic sentiment analysis.

The proposed model consists of AraBERT model, LSTM layer, feedforward neural networks, and output layer as shown in Figure 2. AraBERT is used to capture rich contextual information and LSTM to enhance sequence modeling and retain long-term dependencies within the text data.

Our proposed model incorporates the following layers that work together to capture contextual information from the input text:

- In the initial phase, we employ word embedding from bert-base-arabertv02. These are fed into an AraBERT model, which includes base-arabert. It encompasses 12 Transformer blocks, a hidden layer size of 768, 12 self-attention heads, and a total of 110 M parameters in its pre-trained mode.
- In the subsequent phase, the output of the base-arabert model is passed through an LSTM layer with a Rectified Linear Unit (ReLU) activation function. The LSTM layer consists of 150 units. the LSTM layer enhances sequence modeling and retain long-term dependencies within the text data.
- During the final phase, the last hidden state of the LSTM layer serves as a summary of the sentence's context, encapsulating sentiment-related information. This hidden state transforms a fully connected layer, which maps the LSTM output to sentiment labels. The SoftMax activation function is applied in the output layer to produce a class for the sentiment analysis dataset.

#### 3.7.1 Performance metrics

The performance of a classification system is typically measured by precision, recall, f-score, and accuracy metrics. There are four types of true positives, false positives, true negatives, and false negatives. These are calculated as follows: TP, FP, TN, and FN. While (TN) showed a negative result, it returned a positive result, while (TP) showed a negative result, but it returned a positive result. Positive results are indicated by (TP), while negative results are indicated by (TN).


$$\begin{array}{l} Accuracy=\frac{TP+TN}{TP+FP+TN+FN}. \end{array}$$

$$\begin{array}{l} Precision=\frac{\text{TP}}{\text{TP + FP}} \end{array}$$

$$\begin{array}{l} Recall=\frac{TP}{TP+FN} \end{array}$$

$$\begin{array}{l} F1-score=\frac{2\cdot precision\cdot recall}{precision+recall} \end{array}$$

A confusion matrix is a table used to define classification algorithm performance. A confusion matrix summarizes and visualizes a classification algorithm's performance. Confusion matrices represent percentage from predicted and actual values (Kulkarni et al., 2020).

## 4 Experiments results

### 4.1 Experimental setup

The experiments were conducted using Jupyter Notebook on a machine running Microsoft Windows 10 equipped with an Intel Core i7 CPU and 16 GB of RAM. Multiple evaluation methods, including accuracy, precision, recall, F1-score, and confusion matrix, were employed to assess the efficacy of our models. Transformer models were implemented using PyTorch, while DL models were implemented using Keras, and ML models were implemented using sci-kit-learn. We applied different representation feature methods: TF-IDF, CBOW, SkipGram, and AraBERT. Additionally, various ML models, DL models, and transformer models were applied to four Arabic sentiment analysis datasets: SS2030, ASTC, Main-AHS, and Sub-AHS. The results for each dataset were recorded.

For DL models, the configuration settings were as follows: number of units = 400, learning rate = 0.0005, optimizer = Adam, epochs = 50, and batch size = 32. The settings for the BERT classifier were: hidden size of BERT = 768, hidden size of our classifier = 50, number of labels = 2, optimizer = Adam, learning rate = 0.00005, epochs = 50, and batch size = 32.

Every dataset is divided into 80% training and 20 %testing sets. To train ML, DL, and transformer models, utilize the training set. Models are assessed on a testing set. Each dataset's number of tweets is displayed in Table 1.

**Table 1:** The number of tweets in each dataset.

**Datasets**	**Sets**	**Positive**	**Negative**	**Total**
SS2030	Training set	1,812	1,395	3,207
	Testing set	453	349	802
				4,009
ASTC	Training set	22,810	22,624	45,434
	Testing set	5,703	5,656	11,359
				56,793
Main-AHS	Training set	502	1,118	1,620
	Testing set	126	280	406
				2,026
Sub-AHS	Training set	401	985	1,386
	Testing set	101	246	347
				1,733

### 4.2 The result of SS2030

According to Table 2, we can compare the results of the tested ML, DL, and transformer classifiers for SS2030. The results of ML models show that SVM was more accurate than RF in almost all evaluation measures, achieving 86.41 accuracy and 86.46 precision. RF recorded the second-best performance with an accuracy of 85.66, precision of 85.95, recall of 85.66, and an F1-score of 85.50. KNN had the worst performance, with 80.42 accuracy and an 80.50 F1-score.

**Table 2:** The results of models for SS2030 dataset.

**Approach models**	**Models**	**FS methods**	**Testing performance**			
			**Accuracy**	**Precision**	**Recall**	**F1-score**
ML models	RF	TF-IDF	85.66	85.95	85.66	85.50
	LR	TF-IDF	84.78	84.85	84.78	84.70
	DT	TF-IDF	83.54	83.57	83.54	83.44
	SVM	TF-IDF	86.41	86.46	86.41	86.33
	NB	TF-IDF	82.29	82.71	82.29	82.01
	KNN	TF-IDF	80.42	80.00	80.42	80.50
DL models	LSTM	CBOW	85.04	85.07	85.04	85.06
	GRU	CBOW	85.31	85.23	85.31	85.24
	LSTM	SkipGram	86.92	86.92	86.92	86.83
	GRU	SkipGram	88.53	88.71	88.53	88.44
Transformer model	AraBERT-LSTM	AraBERT	90.40	90.43	90.40	90.41

When comparing the results of DL, SkipGram performed better than CBOW. GRU with SkipGram recorded the best accuracy performance at 88.53 and precision at 88.71. On the other hand, LSTM with CBOW had the lowest values for accuracy at 85.04 and precision at 85.07.

The transformer model performed well and outperformed the other models, achieving the highest performance (accuracy = 90.40, precision = 90.43, recall = 90.4, and F1-score = 90.41), improving performance by 2%.

**Figure 3:**
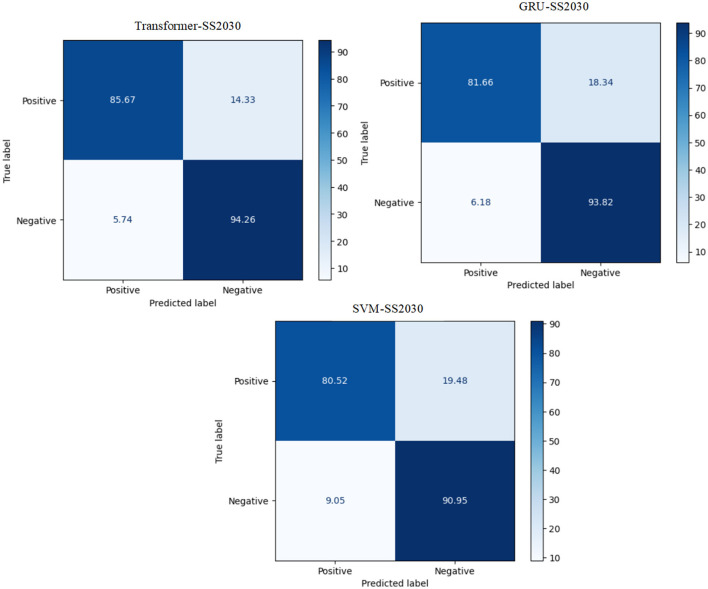
The CM of the best models for SS2030.

The experiment proves that the transformer model recorded the best Arabic sentiment analysis performance. The confusion matrix of the best models, ML, DL, and transformer models for SS2030, is presented in Figure 3. We can see that the transformer model has a high ability to distinguish between class 0 and class 1 compared to SVM and GRU, with 85.67 for TP and 94.26 for TN.

### 4.3 The result of ASTC

According to Table 3, we can compare the results of the tested ML, DL, and transformer classifiers for ASTC. The results of ML models show that RF was more accurate than LR in almost all evaluation measures, achieving 90.77 accuracy and 90.78 precision. SVM recorded the second-best performance with an accuracy of 90.16, precision of 90.26, recall of 90.16, and an F1-score of 90.16. NB had the worst performance, with 85.52 accuracy and an 85.83 F1-score.

**Table 3:** The results of models for ASTC dataset.

**Approach**	**Models**	**FS methods**	**Performance**			
			**Accuracy**	**Precision**	**Recall**	**F1-score**
ML models	RF	TF-IDF	90.77	90.78	90.77	90.76
	LR	TF-IDF	89.39	89.52	89.39	89.38
	DT	TF-IDF	89.21	89.33	89.21	89.20
	SVM	TF-IDF	90.16	90.26	90.16	90.16
	NB	TF-IDF	85.52	85.87	85.52	85.49
	KNN	TF-IDF	88.83	88.83	88.83	88.83
DL models	LSTM	CBOW	90.40	90.58	90.40	90.39
	GRU	CBOW	89.60	89.61	89.60	89.60
	LSTM	SkipGram	92.48	92.52	92.48	92.48
	GRU	SkipGram	91.94	91.94	91.94	91.94
Transformer model	AraBERT-LSTM	AraBERT	93.76	93.77	93.76	93.76

When comparing the results of DL, SkipGram performed better than CBOW. LSTM with SkipGram recorded the best accuracy performance at 92.48 and precision at 92.52. On the other hand, GRU with CBOW had the lowest values for accuracy at 89.60 and precision at 89.61.

The transformer model performed well and outperformed the other models, achieving the highest performance (accuracy = 93.76, precision = 93.77, recall = 93.76, and F1-score = 93.76), improving performance by 1%.

**Figure 4:**
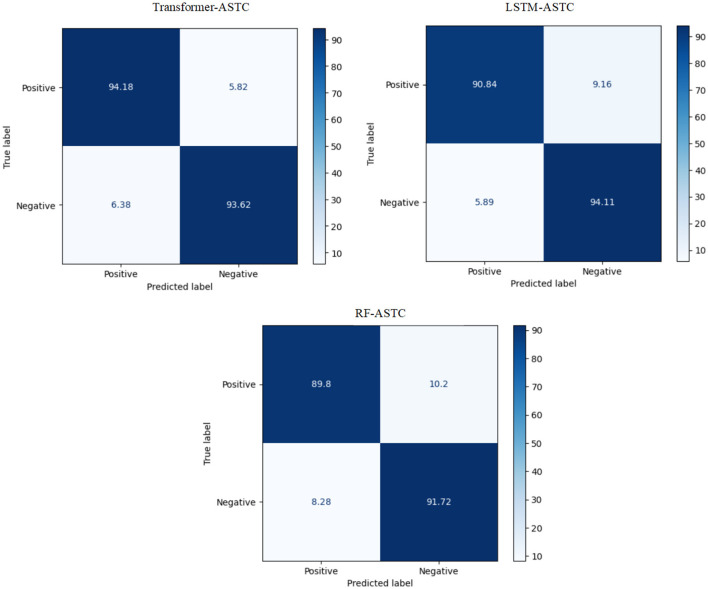
The CM of the best models for ASTC.

The experiment proves that the transformer model recorded the best Arabic sentiment analysis performance. The confusion matrix of the best models, ML, DL, and transformer models for ASTC, is presented in Figure 4. We can see that the transformer model demonstrates a high ability to distinguish between class 0 and class 1, achieving 94.18% true positives (TP) and 93.62% true negatives (TN) compared to SVM and LSTM.

### 4.4 The result of Main-AHS

According to Table 4, we can compare the performance of the ML, DL, and transformer classifiers for Main-AHS. According to ML models, SVM achieved 88.67 accuracy and 88.53 precision, which is more accurate than RF in almost all evaluation measures. In terms of accuracy, precision, recall, and F1-score, RF recorded the second-best performance with 87.90 accuracy, 87.96 precision, and 87.74 F1-score. The worst performance was by NB, with 85.47 accuracy and 85.47 F1-score.

**Table 4:** The results of models for Main-AHS dataset.

**Approach models**	**Models**	**FS methods**	**Performance**			
			**Accuracy**	**Precision**	**Recall**	**F1-score**
ML models	RF	TF-IDF	87.90	87.80	87.90	87.74
	LR	TF-IDF	87.41	87.96	87.41	87.91
	DT	TF-IDF	80.54	80.98	80.54	80.72
	SVM	TF-IDF	88.67	88.53	88.67	88.47
	NB	TF-IDF	85.47	86.63	85.47	85.47
	KNN	TF-IDF	86.41	86.63	86.41	86.02
DL models	LSTM	CBOW	89.31	89.27	89.31	89.35
	GRU	CBOW	88.89	88.63	88.89	88.26
	LSTM	SkipGram	89.45	89.24	89.45	89.27
	GRU	SkipGram	90.15	90.60	90.15	89.73
Transformer model	AraBERT-LSTM	AraBERT	92.61	92.61	92.61	92.61

DL results showed SkipGram to be more effective than CBOW when compared. As a result, LSTM with SkipGram achieved the best accuracy and precision results, with 90.15 and 90.60, respectively. In contrast, GRU with CBOW recorded the lowest accuracy and precision results.

The transformer model performed well and outperformed the other models, achieving the highest performance (accuracy = 92.61, precision = 92.61, recall = 92.61, and F1-score = 92.61), improving performance by 2%.

**Figure 5:**
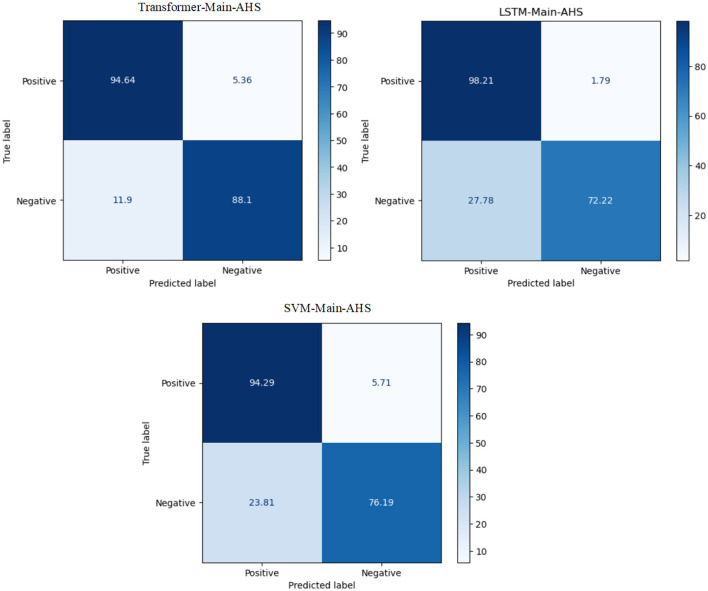
The CM of the best models for Main-AHS.

The experiment proves that the transformer model recorded the best Arabic sentiment analysis performance. The confusion matrix of the best models, ML, DL, and transformer models for Main, is presented in Figure 5. We can see that the transformer model has a high ability to distinguish between class 0 and class 1 compared to SVM and LSTM, with 94.64 for TP and 88.1 for TN.

### 4.5 The result of Sub-AHS

According to Table 5, we can compare the results of the tested ML, DL, and transformer classifiers for Sub-AHS. The results of ML models show that RF was more accurate than NB and DT in all evaluation measures. It achieved 91.93 accuracy and precision. LR recorded the second-best performance in accuracy at 91.35, precision at 91.65, recall at 91.35, and F1-score at 91.01. NB had the worst performance at 87.90 accuracy and 88.23 F1-score.

**Table 5:** The results of models for Sub-AHS.

**Approach models**	**Models**	**FS methods**	**Cross validation performance**			
			**Accuracy**	**Precision**	**Recall**	**F1-score**
ML models	RF	TF-IDF	91.93	91.86	91.93	91.80
	LR	TF-IDF	91.35	91.65	91.35	91.01
	DT	TF-IDF	88.03	88.07	88.03	88.05
	SVM	TF-IDF	90.80	90.95	92.80	90.58
	NB	TF-IDF	87.90	89.44	87.90	88.23
	KNN	TF-IDF	90.80	90.81	90.80	90.6
DL models	LSTM	CBOW	92.52	92.63	92.52	92.41
	GRU	CBOW	92.57	92.36	92.57	92.57
	LSTM	SkipGram	93.08	93.19	93.08	93.12
	GRU	SkipGram	93.37	93.32	93.37	93.32
Transformer model	AraBERT-LSTM	AraBERT	97.12	97.10	97.12	97.10

By comparing the results of DL, SkipGram performed better than CBOW. The best accuracy performance was achieved by GRU with SkipGram at 93.37. LSTM with CBOW recorded the lowest accuracy performance at 92.52.

The transformer model performed well and outperformed other models. It achieved the highest performance (accuracy = 97.12, precision = 97.10, recall = 97.10, and F1-score = 97.12), improving performance by 5%.

**Figure 6:**
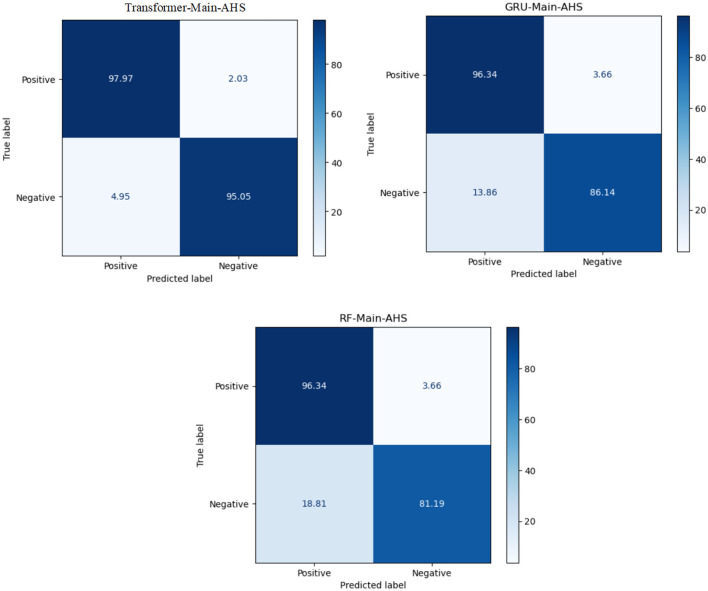
The CM of the best models for Sub-AHS.

The transformer model performed the best in Arabic sentiment analysis. The confusion matrix of the highest-ranking models, ML, DL, and transformer models for SS2030 is presented in Figure 6. We can see that the transformer model can distinguish between class 0 and class 1 rather than SVM and GRU. It has 97.97% for TP and 97.97% for TN.

### 4.6 Discussion

This section presents the comparison between the best models for each dataset. The comparison performance is based on four methods: accuracy, precision, recall, and F1-score.

**Figure 7:**
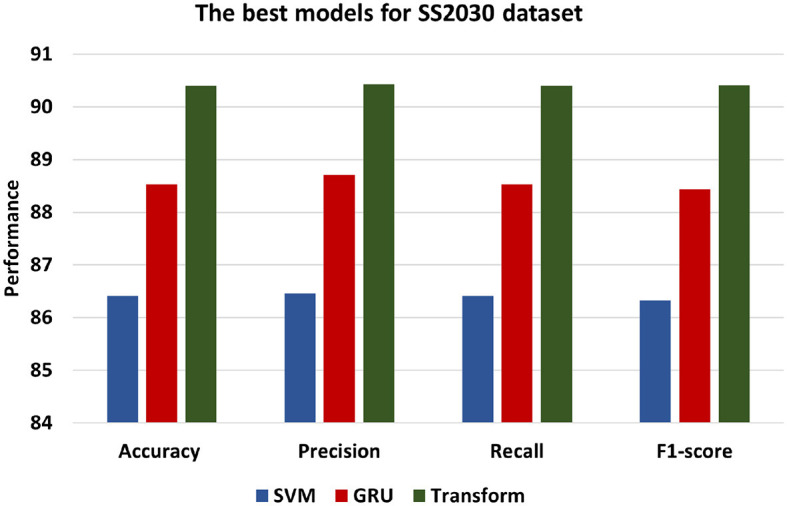
The best models for SS2030 dataset.

Figure 7 presents the best models for SS2030 datasets which are ML models is SVM, DL is GRU and and Transformer. In contrast, the Transformer model demonstrates the most superior performance among the models, achieving an accuracy of 90.40. This signifies a higher level of accuracy compared to both the SVM and GRU models. Additionally, with a precision of 90.43, the Transformer model showcases an enhanced ability to accurately classify positive instances in comparison to the other models. The recall of 90.40 indicates that the Transformer model excels in identifying positive statements, and F1-score of 90.41 reflects a balanced performance. Comparatively, the GRU model performs marginally better than the SVM model, exhibiting an accuracy of 88.50, Precision of 88.71, Recall of 88.53, and F1-score of 88.44. This indicates that the GRU model excels in classifying instances with greater performance than the SVM model.

**Figure 8:**
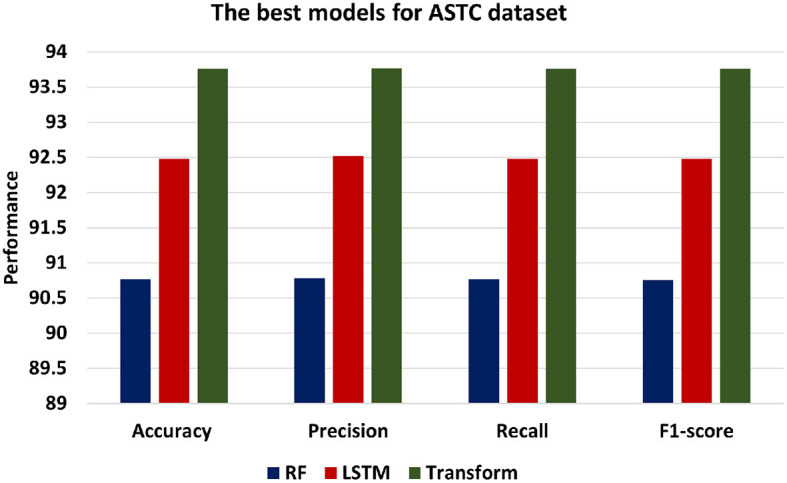
The best models for ASTC dataset.

Figure 8 presents the best models for ASTC datasets which are ML models is RF, DL is LSTM and Transformer. Transformer model achieves the highest level of accuracy among other models, achieving 93.76. In addition, the Transformer model's precision of 93.77 shows that it is more accurate at classifying positive instances than the other models. Transformer's recall of 93.76 demonstrates its success in identifying positive statements. F1-score of 93.76 indicates a balanced performance. Comparatively, the LSTM model performs marginally better than the RF model, exhibiting an accuracy of 92.48, Precision of 92.52, Recall of 92.48, and F1-score of 92.48.

**Figure 9:**
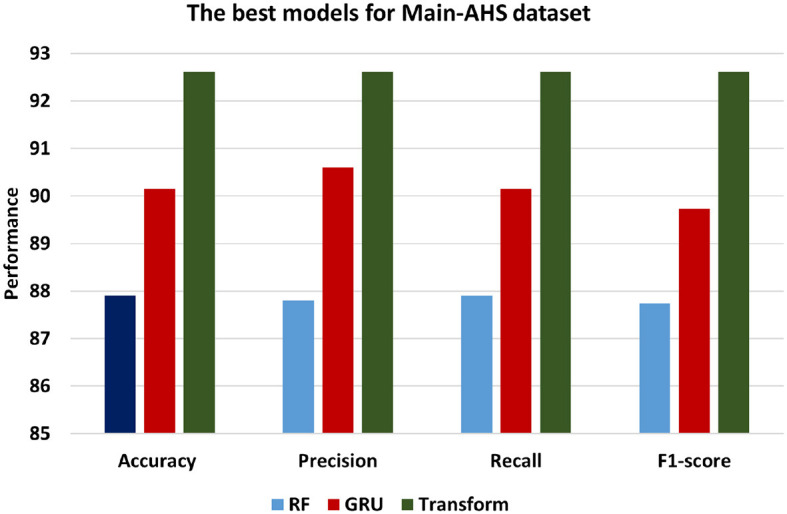
The best models for Main-AHS dataset.

Figure 9 presents the best models for Main-AHS dataset which are ML models is RF, DL is GRU and BERT Transformer. In contrast, the Transformer model demonstrates the most superior performance among the three models, achieving an accuracy of 92.61. This signifies a higher level of accuracy compared to both the SVM and GRU models. Additionally, with a precision of 92.61, the Transformer model showcases an enhanced ability to accurately classify positive instances in comparison to the other models. The recall of 92.61 indicates that the Transformer model excels in identifying positive statements, and F1-score of 92.61 reflects a balanced performance. Comparatively, the GRU model performs marginally better than the SVM model, exhibiting an accuracy of 90.15, Precision of 90.6, Recall of 90.15, and F1-score of 89.73. This indicates that the GRU model excels in classifying instances with greater performance than the RF model.

**Figure 10:**
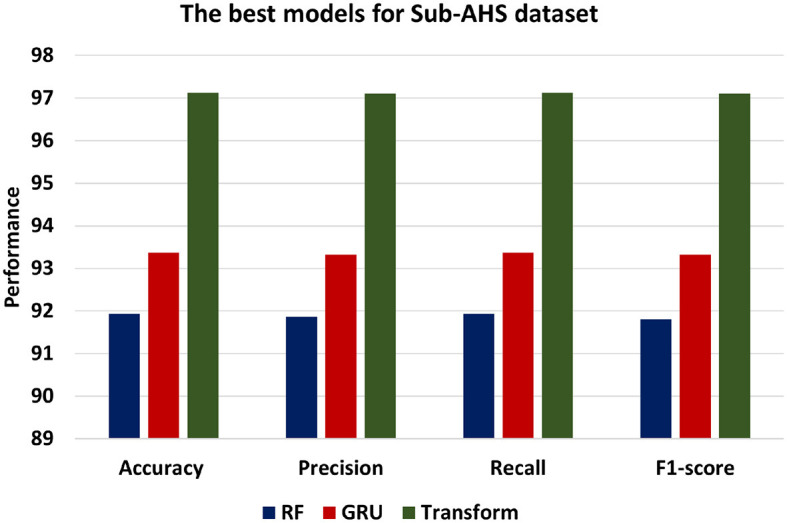
The best models for Sub-AHS dataset.

Figure 10 presents the best models for Sub-AHS dataset which are ML models is RF, DL is LSTM and BERT Transformer. Transformer achieves the highest level of accuracy among other models, achieving 97.12. In addition, the Transformer model's precision of 97.10 shows that it is more accurate at classifying positive instances than the other models. Transformer's recall of 97.12 demonstrates its success in identifying positive statements. F1-score of 97.1 indicates a balanced performance. Comparatively, the GRU model performs marginally better than the RF model, exhibiting an accuracy of 93.37, Precision of 93.32, Recall of 93.37, and F1-score of 93.32.

Overall, the results indicate that the Transformer model outperforms both the ML and DL models in terms of accuracy, precision, recall, and F1-score. It achieves the highest level of performance.

### 4.7 Comparison with the state-of-the-art

Table 6 shows the comparison results of our work with the state-of-the-art. The proposed model uses the base AraBERT version 2, the results showed that the transformer model achieved the highest accuracy compared with the other research. SS2030 is a recent dataset that was published in 2020 (Alyami and Olatunji, 2020), the authors applied SVM with 89.83 accuracy. Our work applied a transformer model that achieved 90.40 accuracy. The ASTC was used in Almouzini et al. (2019), Bolbol and Maghari (2020), and Saleh et al. (2022). In Almouzini et al. (2019) was applied BiLSTM with 90 of accuracy, in Saleh et al. (2022) was applied the stacking DL with 92 of accuracy, in Bolbol and Maghari (2020) was applied LR with 91 of accuracy. Our work applied a transformer model with the highest accuracy at 93.76. Main-AHS was applied in Alayba et al. (2018) and Almouzini et al. (2019) with CNN and BiLSTM that recorded 91 accuracy. Our work applied a transformer model with the highest accuracy at 92.61. Main-AHS was applied in Almouzini et al. (2019) with BiLSTM that recorded 82 accuracy. Our work applied a transformer model with the highest accuracy at 97.12.

**Table 6:** Comparison with the state-of-the-art.

**References**	**Models**	**Word embedding**	**Datasets**	**Performance**
Alyami and Olatunji (2020)	SVM	Ngrams	SS2030	Accuracy = 89.83
Alayba et al. (2018)	CNN	Word2Vec	Main-AHS	Accuracy = 91
Almouzini et al. (2019)	BiLSTM	Word2Vec	Main-AHS	Accuracy = 91
	BiLSTM	Word2Vec	Sub-AHS	Accuracy = 82
	BiLSTM	Word2Vec	ASTC	Accuracy = 90
Saleh et al. (2022)	Stacking DL	CBOW	ASTC	Accuracy = 92
Bolbol and Maghari (2020)	LR	TF-IDF	ASTC	Accuracy = 91
Our work	Transformer model	AraBERT	SS2030	Accuracy = 90.40
		AraBERT	ASTC	Accuracy = 93.76
		AraBERT	Main-AHS	Accuracy = 92.61
		AraBERT	Sub-AHS	Accuracy = 97.12

## 5 Conclusion

In this paper, we propose a model for Arabic sentiment analysis based on four datasets. The suggested model is based on a transformer model and LSTM, named AraBERT-LSTM. The model includes the AraBERT model, an LSTM layer, feedforward neural networks, and an output layer. Firstly, pre-processing techniques, such as stemming, normalization, tokenization, and stop word removal, were applied to clean the text. Additionally, multiple word embeddings, including CBOW and Skip-Gram, as well as AraBERT, were employed to represent vectors. The proposed model is compared with both ML and DL models. The results of the study reveal that the proposed model outperforms several established state-of-the-art approaches when tested on relevant datasets, resulting in substantial performance improvements. For SS2030, the proposed model achieved an accuracy of 90.40 and an F1-score of 90.41. In the case of ASTC, the proposed model demonstrated an accuracy of 93.76 and an F1-score of 93.76. Regarding Main-AHS, the proposed model attained an accuracy of 92.61 and an F1-score of 92.61. Lastly, for Sub-AHS, the proposed model achieved an accuracy of 97.12 and an F1-score of 97.12. In our future work, we intend to explore and incorporate additional cutting-edge methods to further enhance our research and address the ever-evolving challenges in the field.

## Data availability statement

The original contributions presented in the study are included in the article/supplementary material, further inquiries can be directed to the corresponding author.

## Author contributions

WA: Data curation, Funding acquisition, Writing – original draft, Writing – review & editing. HS: Investigation, Methodology, Visualization, Writing – original draft, Writing – review & editing. AH: Methodology, Writing – original draft, Writing – review & editing. NE-R: Data curation, Investigation, Validation, Writing – original draft. AA: Investigation, Software, Writing – original draft, Writing – review & editing. AE: Data curation, Validation, Writing – original draft, Writing – review & editing. SM: Investigation, Methodology, Software, Writing – original draft, Writing – review & editing.
